# Ramp Tear among Patients Undergoing Arthroscopic Anterior Cruciate Ligament Reconstruction in a Tertiary Care Centre

**DOI:** 10.31729/jnma.8235

**Published:** 2023-08-31

**Authors:** Amit Joshi, Santosh Nepal, Subhash Regmi, Ishor Pradhan, Nagmani Singh, Bibek Basukal, Rohit Bista, Rajiv Sharma

**Affiliations:** 1 Department of Orthopedics, B&B Hospital Pvt. Ltd., Gwarko, Lalitpur, Nepal

**Keywords:** *anterior cruciate ligament injuries*, *anterior cruciate ligament reconstruction*, *arthroscopy*

## Abstract

**Introduction:**

Ramp tear is a specific injury that affects the posterior horn of the medial meniscus and its meniscosynovial or meniscocapsular attachments. The actual prevalence of ramp lesion is unknown due to the high probability of misdiagnosis or underdiagnosis caused by the low sensitivity of imaging modalities and poor visualization during arthroscopy. This study aimed to find out the prevalence of ramp tear among patients undergoing arthroscopic anterior cruciate ligament reconstruction in a tertiary care centre.

**Methods:**

A descriptive cross-sectional study was conducted among patients undergoing arthroscopic anterior cruciate ligament reconstruction after getting ethical approval from the Institutional Review Committee. Data from 1 March 2019 to 31 December 2022 was collected between 1 May 2023 to 30 May 2023 from medical records. The study included all patients who underwent arthroscopic anterior cruciate ligament reconstruction. Patients with a previous history of medial meniscus injury or repair and undergoing revision anterior cruciate ligament reconstruction were excluded. Convenience sampling method was used. The point estimate was calculated at a 95% Confidence Interval.

**Results:**

Out of 412 patients who underwent arthroscopic anterior cruciate ligament reconstruction, 53 (12.86%) (9.63-16.09, 95% Confidence Interval) had ramp tears. The mean age of patients with ramp tears was 28.64±7.57 years. Among 53 patients, 42 (79.24%) were male and 11 (20.75%) were female.

**Conclusions:**

The prevalence of ramp tears in patients undergoing arthroscopic anterior cruciate ligament reconstruction in a tertiary care centre was found to be lower than other studies done in other international studies.

## INTRODUCTION

Ramp tears are a specific form of injury that affects the posterior horn of the medial meniscus and its meniscosynovial or meniscocapsular attachments.^1^ These injuries are often linked with anterior cruciate ligament (ACL) injuries because excessive anterior tibial subluxation caused by ACL tear stimulates semimembranosus tendon contraction, putting tension on the posteromedial articular capsule and trapping the meniscus between femur and tibia, resulting in ramp tears.^2,3^ These lesions contribute to knee instability because of their association with increased anterior tibial translation, rotational laxity, and excessive rotational knee motion, all of which have a negative impact on knee stability.^4^

The actual prevalence of ramp lesions is unknown due to the high probability of misdiagnosis or underdiagnosis caused by the low sensitivity of imaging modalities and poor visualization during arthroscopy.^5^

This study aimed to find out the prevalence of ramp tears among patients undergoing arthroscopic anterior cruciate ligament reconstruction in a tertiary care centre.

## METHODS

A descriptive cross-sectional study was conducted among patients undergoing arthroscopic ACL reconstruction after obtaining ethical approval from the Institutional Review Committee (IRC) of B&B Hospital, Gwarko, Lalitpur, Nepal (Reference number: B&BIRC-22-50). Data from 1 March 2019 to 31 December 2022 was collected between 1 May 2023 to 30 May 2023 from medical records. The study included all patients who underwent arthroscopic ACL reconstruction. Patients with a previous history of medial meniscus injury or repair and undergoing revision ACL reconstruction and those with incomplete records were excluded. Convenience sampling method was used. The sample size was calculated by using the following formula:


$$\begin{array}{ll} \text{n} & ={\text{Z}}^{2}\times \frac{\text{p}\times \text{q}}{{\text{e}}^{\text{2}}}\\ & =1.96^{2}\times \frac{0.50\times 0.50}{0.05^{2}}\\ & =385 \end{array}$$

Where,

n = minimum required sample size
Z = 1.96 at 95% Confidence Interval (CI)
p = prevalence taken as 50% for maximum sample size calculation
q = 1-p
e = margin of error, 5%

The minimum sample size was calculated as 385. However, a total of 412 patients were included in the study.

The grades of the pivot shift test based on clinical examination were also recorded. Before arthroscopic ACL reconstruction, a comprehensive diagnostic arthroscopy of all knee compartments was conducted with the patient in the standard arthroscopy position using anteromedial and anterolateral portals.^6^

The ramp lesion was considered to be present if there was a longitudinal tear in the peripheral attachment of the posterior horn of the medial meniscus (red-red zone or meniscocapsular junction) extending longitudinally for <3 cm.^7^ The ramp tears were classified into five subtypes: type 1- meniscocapsular tear, type 2- partial superior tear, type 3 partial inferior tear or hidden type, type 4- complete tear, and type 5- double tear.^8^

Data were entered and analyzed using Microsoft Excel 2016. The point estimate was calculated at a 95% Confidence Interval.

## RESULTS

Among 412 patients who underwent arthroscopic ACL reconstruction, 53 (12.86%) patients (9.63-16.09, 95% CI) had ramp tears. The mean age of patients with ramp tears was 28.64±7.57 years. Among 53 patients, 42 (79.24%) were male, and 11 (20.75%) were females. Twenty-five (47.2%) patients had road traffic accidents, and 19 (35.8%) had sports-related injuries (Table 1).

**Table 1:** Baseline characteristics of patients with ramp tear (n= 53).

Parameters	n (%)
**Mechanism of injury**	
Road traffic accident	25 (47.16)
Sports	19 (35.84)
Fall	9 (16.98)
**Delay in presentation**	
<1 month	5 (9.43)
1-3 months	7 (13.20)
3-6 months	9 (16.98)
6 months-1 year	11 (20.75)
>1 year	21 (39.62)
**Pivot shift**	
Grade 1	12 (22.64)
Grade 2	31 (58.49)
Grade 3	10 (18.86)
**Types of ramp tear**	
Type 1	27 (50.94)
Type 2	3 (5.66)
Type 3	6 (11.32)
Type 4	12 (22.64)
Type 5	5 (9.43)

## DISCUSSION

In our study, the prevalence of ramp tears in patients undergoing arthroscopic ACL reconstruction was 53 (12.86%). The finding was lower than reported in other international studies, which was around 16-24%.^3,7^ A study conducted in France, including 3214 patients with ACL injured knee undergoing arthroscopic ACL reconstruction, found that the prevalence of ramp tear was around 24%.^3^ Similarly, another study conducted in China, including 868 patients undergoing arthroscopic ACL reconstruction, found that the prevalence of ramp tears was around 17%.^7^ The exact reason for the lower prevalence of ramp tears in this study could not be identified. However, it could be due to the lower prevalence of grade 3 pivot shift among our included participants. It has been suggested that there could be an association of increased dynamic rotatory laxity with the occurrence of ramp tears.^4^ In this study, the prevalence of grade 3 pivot shift was 18.86%. The finding was lower compared to that reported in international studies, which was around 25-47%.^4,8^

This study also identified that the most common type of ramp tear was type 1 (50.94%). The finding was similar to that reported in other international studies, where the type 1 tear was most common, with a prevalence of around 48-60%.^8,9^ A study conducted in France involving 334 ramp tears observed that the type 1 tear was the most common type, with a prevalence of around 48%.^8^ Similarly, another study conducted in Iran involving 93 ramp tears observed that the type 1 tear was the most common type with a prevalence of around 60%.^9^ This suggests that type 1 tear is the most common type of ramp tear.

Some studies have suggested that a longer duration from injury to operation may lead to an increased occurrence of ramp tears among patients with ACL injuries.^3,7^ In this study, the majority of the patients with ramp tears (around 60%) presented after 6 months of injury. The finding was similar to that reported in other international studies, in which most of their patients presented after six months of injury.^7,8^ This suggests that delay in treatment in patients with ACL injury may lead to ramp tear.

This study has some limitations. As this is a descriptive cross-sectional study, there may be some inherent biases. The outcomes of this study cannot be generalized to all patients with ACL injury as only those undergoing arthroscopic ACL reconstruction were included. The exact causal relationship between ACL deficiency and the occurrence of ramp tear could not be established. However, this study provides the contextual prevalence of ramp tears in patients with ACL injury undergoing arthroscopic ACL reconstruction. This suggests that sports surgeons need to be more vigilant of such injuries while treating patients with ACL injuries.

## CONCLUSIONS

The prevalence of ramp tears in patients undergoing arthroscopic ACL reconstruction in a tertiary care centre was lower than other studies. However, sports surgeons need to be more vigilant of such injuries while treating patients with ACL injuries. Future studies with more suitable designs could establish a causal relation between ACL injury and the occurrence of ramp tears.
